# ﻿Comparative mitogenomic analysis of three bugs of the genus *Hygia* Uhler, 1861 (Hemiptera, Coreidae) and their phylogenetic position

**DOI:** 10.3897/zookeys.1179.100006

**Published:** 2023-09-08

**Authors:** Shijun Wang, Xiaofei Ding, Wenbo Yi, Wanqing Zhao, Qing Zhao, Hufang Zhang

**Affiliations:** 1 College of Plant Protection, Shanxi Agriculture University, Jinzhong 030800, Shanxi, China Shanxi Agriculture University Jinzhong China; 2 Department of Biology, Xinzhou Teachers University, Xinzhou 034000, Shanxi, China Xinzhou Teachers University Xinzhou China

**Keywords:** Coreoidea, evolutionary relationships, mitogenome, phylogeny

## Abstract

*Hygia* Uhler, 1861 is the largest genus in the bug family Coreidae. Even though many species of this genus are economically important, the complete mitogenomes of *Hygia* species have not yet been reported. Therefore, in the present study, the complete mitogenomes of three *Hygia* species, *H.lativentris* (Motschulsky, 1866), *H.bidentata* Ren, 1987, and *H.opaca* (Uhler, 1860), are sequenced and characterized, and submitted in a phylogenetic analysis of the Coreidae. The results show that mitogenomes of the three species are highly conserved, typically with 37 genes plus its control region. The lengths are 16,313 bp, 17,023 bp, and 17,022 bp, respectively. Most protein-coding genes (PCGs) in all species start with the standard codon ATN and terminate with one of three stop codons: TAA, TAG, or T. The tRNAs secondary structures of all species have a typical clover structure, except for the *trnS1* (AGC) in *H.bidentata*, which lacks dihydrouridine (DHU) arm that forms a simple loop. Variation in the length of the control region led to differences in mitochondrial genome sizes. The maximum-likelihood (ML) and Bayesian-inference (BI) phylogenetic analyses strongly supported the monophyly of *Hygia* and its position within Coreidae, and the relationships are ((*H.bidentata* + (*H.opaca* + (*H.lativentris + Hygia* sp.))). The results provide further understanding for future phylogenetic studies of Coreidae.

## ﻿Introduction

The mitogenome of insects usually comprises a double-stranded circular DNA molecule ranging from 14 to 18 kb in size, containing 37 genes (22 transfer RNAs, 13 protein-coding genes, 2 ribosomal RNAs) and a control region, which is consistent with the most typical insect mitochondrial genome, namely *Drosophilayakuba* Burla (Brown et al. 1979; Clary and Wolstenholme 1985; Boore 1999; Curole and Kocher 1999; Galtier et al. 2009; Cameron 2014). It has maternal inheritance, low sequence recombination, and rapid evolution. Because of these characteristics and the rapid development of high-throughput sequencing technology, the analysis of insect mitogenomes has been widely used in taxonomy, population genetics, evolutionary biology, phylogenetics, and biogeographic studies, covering all orders of insects (Hua et al. 2008; Cameron 2014; Yang et al. 2018; Du et al. 2019; Kieran 2020; Zheng et al. 2020; Dong et al. 2021; Manchola et al. 2021). Notably, phylogenetic analysis based on the mitochondrial whole genome has a higher resolution than phylogenetic trees based on partial gene fragments.

Coreidae, well known for its odious defensive or alarm pheromones, is the largest family of Coreoidea, including 481 genera and 2,584 species. It is widely distributed worldwide (Aldrich and Blum 1978; Leal et al. 1994; Coreoidea Species Files 2022), and several of its species, such as *Cletuspunctiger* (Dallas, 1852) and *Leptoglossusoccidentalis* Heidemann, 1910, are major insect pests that can cause huge economic losses (Mitchell 2000). Currently, Coreidae comprises four recognized subfamilies: Coreinae, Hydarinae, Meropachyinae, and Pseudophloeinae (Schuh and Weirauch 2020; Coreoidea Species Files 2022). There are some related studies on the coreoid phylogenetic hypotheses. Using cladistic analysis based on morphological characteristics, Li (1996) found that major relationship was: Pseudophloeinae + (Hydarinae + (Rhopalidae + (Alydidae + (Coreinae + Meropachyinae)))). Later, Li (1997) ran morphological analysis on the Coreidae, which supported the paraphyly of Coreinae and showed the relationship among Pseudophloeinae + (Hydarinae + (Coreinae + Meropachyinae)). Other less comprehensive studies have also shown the Pseudophloeinae as an early diverging lineage within Coreidae (Ahmad and Shadab 1975; Ahmad 1979). Owing to limited taxon sampling, a mitochondrial genome analysis by Zhao et al. (2018) suggested that Coreidae was nonmonophyletic, and Pseudophloeinae and Hydarinae were closer to Alydidae than to other coreid subfamilies. The phylogenomic analysis based on ultraconserved element (UCE) loci by Forthman et al. (2019, 2020) showed that the Coreidae were not monophyletic, and the position of the Hydarinae was unstable depending on which analytical approach was used. Specifically, the result of the maximum-likelihood tree was (Hydarinae + Micrelytrinae) + (Alydidae + Pseudophloeinae), while that of the summary coalescent trees was Hydarinae + (Micrelytrinae + (Alydidae + Pseudophloeinae)). Forthman et al. (2022) expanded upon the taxon sampling to investigate relationships among and within the Alydidae, Hydarinae, and Pseudophloeinae using UCEs, and the results robustly corroborated an Alydidae + Hydarinae + Pseudophloeinae clade and resolved the position of Hydarinae as the sister group to a clade consisting of a paraphyletic Alydidae and Pseudophloeinae. The phylogenetic analysis using mitochondrial genes by Dong et al. (2022) also showed similar results.

**Figure 1.**
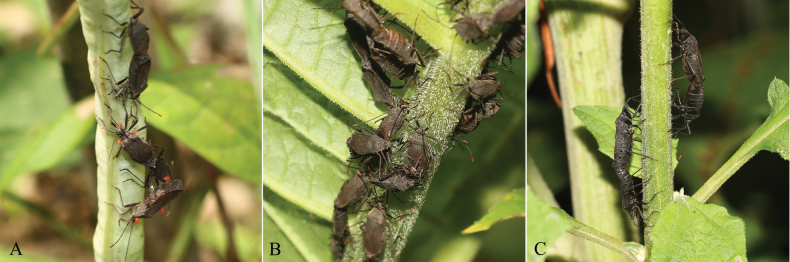
Photographs of *Hygia* species feeding on three host plants in Guizhou, China **A***H.opaca* on *Phaseolusvulgaris* L. **B***H.lativentris* on *Rheumpalmatum* L. **C***H.bidentata* on *Himalaielladeltoidea* (DC.) Raab-Straube.

*Hygia* Uhler, 1861 is a larger genus in the family Coreidae. It is widely distributed in the Oriental and Palearctic regions. The genus includes 10 subgenera and 118 known species worldwide. At present, 26 species in three subgenera are known to be distributed in China (Brailovsky and Barrera 2006; Dolling 2006). The *Hygia* species are black, often with yellow or brown-yellow spots, which can cause identification difficulties. Many species of this genus are economically important, mainly harming plants such as Solanaceae, Polygonaceae, Asteraceae, and Fabaceae. Moreover, most of the species present clustered feeding (Hsiao 1977; Yin and Xiong 1985) (Fig. 1). Most of the previous studies on *Hygia* focused on its morphological and physiological characteristics, as well as some biological characteristics (Hsiao 1977; Noge et al. 2015). So far, only the partial mitochondrial sequence and nuclear DNA sequences of a few *Hygia* species are available in the GenBank. Therefore, it is necessary to analyze more mitogenome sequences of *Hygia* species to better understand the phylogenetic relationships of the genus.

In the present study, we newly sequenced, annotated, characterized, and compared the complete mitogenomes of *H.lativentris* (Motschulsky, 1866), *H.bidentata* Ren, 1987, and *H.opaca* (Uhler, 1860) in detail to reveal the mitogenomic characteristics of the genus *Hygia* and reconstruct the phylogenetic relationships of *Hygia* within the Coreidae with the existing data. Furthermore, we provide more taxon sampling data from the perspective of mitogenomes, which can be used to infer a higher level of evolutionary history later.

## ﻿Materials and methods

### ﻿Sample collection and DNA extraction

Chinese *Hygia* specimens of *H.lativentris*, *H.bidentata*, and *H.opaca* were collected by sweeping in July 2021 from Kuankuoshui National Natural Reserves in Guizhou Province, Zilinshan National Forest Park, Qiannan Autonomous Prefecture, and Jinxiu County in Guangxi Province, respectively. All specimens were placed in ethanol (95%) and stored in −20 °C freezers at the Shanxi Agricultural University (SAU), Shanxi, China. The DNA was extracted from the thorax or leg tissues of individual adults using the Genomic DNA Extraction Kit (Sangon Biotech, Shanghai, China) following the manufacturer’s protocol.

### ﻿Sequencing, assembly, annotation, and sequence analyses

The whole mitogenomes of the three species were sequenced separately using the Illumina NovaSeq platform (Personalbio, Shanghai, China) using a 400-bp insert size and paired-end 150-bp sequencing strategy. Each species’ assembled full-length mitochondrial genome features were annotated using Geneious v. 8.1.4 software (Kearse et al. 2012). Using Open Reading Frame Finder (http://www.ncbi.nlm.nih.gov/gorf/gorf.html) on the NCBI website, the 13 PCGs were predicted based on the invertebrate mitochondrial genetic codes. MEGA 11 (Tamura et al. 2021) was used to translate the nucleotide compositions. The tRNA sequences were identified using MITOS (http://mitos.bioinf.uni-leipzig.de/index.py/) (Bernt et al. 2013) and tRNAscan-SE (http://lowelab.ucsc.edu/tRNAscan-SE/) (Lowe and Eddy 1997). The CG View Server v. 1.0 (Grant and Stothard 2008) was used to draw the circular mitogenomes maps. The formulas AT-skew = (A – T) / (A + T) and GC-skew = (G – C) / (G + C) were used to calculate the AT skew and CG skew (Perna and Kocher 1995). The nucleotide composition, codon usage, and relative synonymous codon usage (RSCU) of the three species were analyzed using PhyloSuite v. 1.1.2 (Zhang et al. 2020). The synonymous substitutions (Ks), nonsynonymous substitutions (Ka), and the Ka/Ks ratios of the 13 PCGs of the *Hygia* species were calculated using DnaSP v. 5.10.01 (Librado and Rozas 2009). The tandem repeats of the control region were identiﬁed using the Tandem Repeats Finder web server (http://tandem.bu.edu/trf/trf.html) (Benson 1999). The mitogenomes of *H.lativentris*, *H.bidentata*, and *H.opaca* were submitted to GenBank under the accession numbers OP837484, OP837485, and OP837486, respectively.

### ﻿Phylogenetic analyses

The phylogenetic relationships within Coreidae were analyzed using the three newly sequenced *Hygia* mitogenomes and another 21 mitogenomes of Coreidae. *Dicranocephalusagilis* (Scopoli, 1763) was used as an outgroup (Table 1). Based on the amino acid sequences, all extracted PCGs were aligned in MEGA11 (Tamura et al. 2021) and then spliced in SequenceMatrix v. 1.7.8 (Vaidya et al. 2011). The best partitioning scheme and best-fit substitution model were selected using PartitionFinder v. 2.0 (Lanfear et al. 2017), and the Bayesian-inference (BI) and maximum-likelihood (ML) methods were used for the phylogenetic analyses of the dataset comprising the 13 PCGs. The BI and ML analyses were performed using MrBayes v. 3.2.7a (Ronquist et al. 2012) and IQ-TREE v. 1.6.10 (Nguyen et al. 2015), respectively. The BI analysis was conducted using two simultaneous Markov chain Monte Carlo (MCMC) runs of 10,000,000 generations with sampling every 1,000 generations, and the first 25% of the trees were discarded as burn-in. ML analysis was conducted using 1,000 bootstrap replicates.

**Table 1.** Mitogenome information used in the present study.

Family	Subfamily	Species	GenBank No.	References
Stenocephalidae		*Dicranocephalusagilis*	JQ910990	Li et al. 2017
Coreidae	Pseudophloeinae	*Gralliclavahorrens*	MW619671	Ye et al. 2022
Coreidae	Hydarinae	*Hydarellaorientalis*	MW619672	Ye et al. 2022
		*Hydaropsislongirostris*	NC_012456	Hua et al. 2008
Coreidae	Coreinae	*Cloresmuspulchellus*	NC_042806	Liu et al. 2019
		*Enoplopssibiricus*	MW619678	Ye et al. 2022
		*Derepteryxlunata*	NC_042807	Liu et al. 2019
		*Mictistenebrosa*	NC_042811	Liu et al. 2019
		*Anoplocnemiscurvipes*	NC_035509	Unpublished
		*Cletuspunctiger*	NC_050997	Zhang et al. 2019a
		*Notopteryxsoror*	NC_037376	Jiang 2017
		*Pseudomictisbrevicornis*	NC_042814	Liu et al. 2019
		*Cletomorpharaja*	NC_063143	Ye et al. 2022
		*Leptoglossusmembranaceus*	NC_042809	Liu et al. 2019
		*Helcomeriaspinosa*	MW619674	Ye et al. 2022
		*Homoeocerusunipunctatus*	MW619675	Ye et al. 2022
		*Hygia* sp.	MW619679	Ye et al. 2022
		*Manocoreus* sp.	MW619724	Ye et al. 2022
		*Petillopsiscalcar*	MW619673	Ye et al. 2022
		*Physomerus* sp.	MW619681	Ye et al. 2022
		*Sinodasynus* sp.	MW619676	Ye et al. 2022
		*Notobitusmontanus*	NC_065112	Unpublished
		*Hygialativentris*	OP837484	This study
		*Hygiabidentata*	OP837485	This study
		*Hygiaopaca*	OP837486	This study

## ﻿Results

### ﻿Mitogenomes features

**Figure 2.**
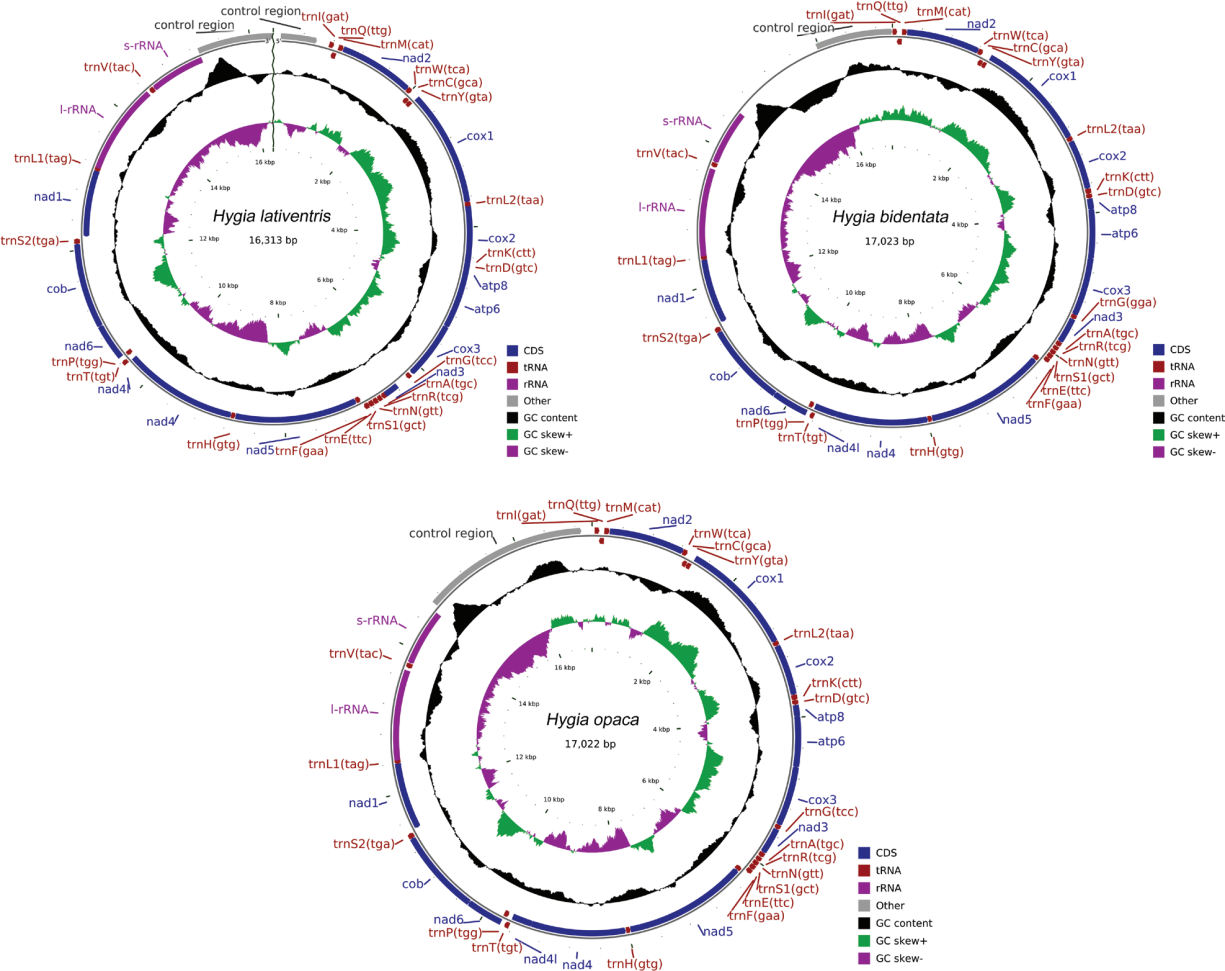
Mitogenome maps of *Hygialativentris*, *H.bidentata*, and *H.opaca*.

The mitogenomes lengths of *H.lativentris*, *H.bidentata*, and *H.opaca* were 16,313 bp, 17,023 bp, and 17,022 bp, respectively (Fig. 2, Suppl. material 1: table S1). Similar to that of many insects, the mitogenomes of the three *Hygia* species were double-stranded loops, with 13 PCGs, 12 tRNAs, 2 rRNAs, and a control region. The gene structure of this genus was in the same direction and order as the hypothesized mitochondrial genes of ancestral insects without rearrangement: 23 genes (containing 14 tRNAs and nine PCGs) were located on the J-strand and 17 genes (containing eight tRNAs, four PCGs, and two rRNAs) were located on the N-strand (Clary and Wolstenholme 1985).

The complete mitogenomes of these three *Hygia* species had a strong A+T base composition bias relative to G+C (76.1–77.3%; mean = 76.73%). The A+T base compositions of *H.lativentris*, *H.bidentata*, and *H.opaca* were 76.8% (A: 42.9%, T: 33.9%, G: 9.7%, C: 13.4%), 77.3% (A: 44.1%, T: 33.2%, G: 9.2%, C: 13.5%), and 76.1% (A: 43.6%, T: 32.5%, G: 9.6%, C: 14.3%), respectively. All three species had positive AT skew values (ranging from 0.117 to 0.146, mean = 0.134) and negative GC-skew values (ranging from −0.197 to −0.159, mean = −0.182) (Suppl. material 1: table S2). They have similar intergenic spacers (ranging from 1–24 bp in size) and gene overlaps (ranging from 1–8 bp). The longest intergenic spacers were all located between *trnS2* and *nad1*, and their lengths of them were 21 bp in *H.lativentris*, 24 bp in *H.bidentata*, and 19 bp in *H.opaca*. The length of the longest overlap was 8 bp, located between *trnW* and *trnC*. All three species had two identical 7 bp gene overlaps, including *atp8*-*atp6* and *nad4*-*nad4l* (Suppl. material 1: table S1).

### ﻿Protein coding genes and codon usage

The 13 PCGs of *H.lativentris*, *H.bidentata*, and *H.opaca* were 11,043 bp, 11,046 bp, and 11,043 bp long, accounting for 67.7%, 64.9%, and 64.9% of the total sequences, respectively (Suppl. material 1: table S1). Among the 13 PCGs, the largest and smallest genes were *nad5* (1,713 bp) and *atp8* (ranging from 156 to 159 bp). Except for the *cox1*, which starts with a non-traditional start codon TTG, all other genes started with the standard codon ATN (ATG, ATT, ATA, and ATC) (Suppl. material 1: table S2). The start codons of 11 PCGs (*nad1*, *nad2*, *nad3*, *nad4*, *nad4l*, *nad5*, *cox1*, *cox2*, *cox3*, *atp6*, and *cytb*) in the three species were consistent. However, the start codons of *atp8* and *nd6* were ATT and ATA, respectively, in both *H.bidentata* and *H.opaca*, while the start codons of *atp8* and *nad6* were ATC and ATT, respectively, in *H.lativentris*. Three types of stop codons occurred in the 13 PCGs: TAA, TAG, and T. Except for *cox1*, *cox2*, and *cox3*, which used T codons – a common feature in insects – the other PCGs finished with a complete TAN codon. The stop codon TAA is more frequent than TAG in all three mitogenomes.

**Figure 3.**
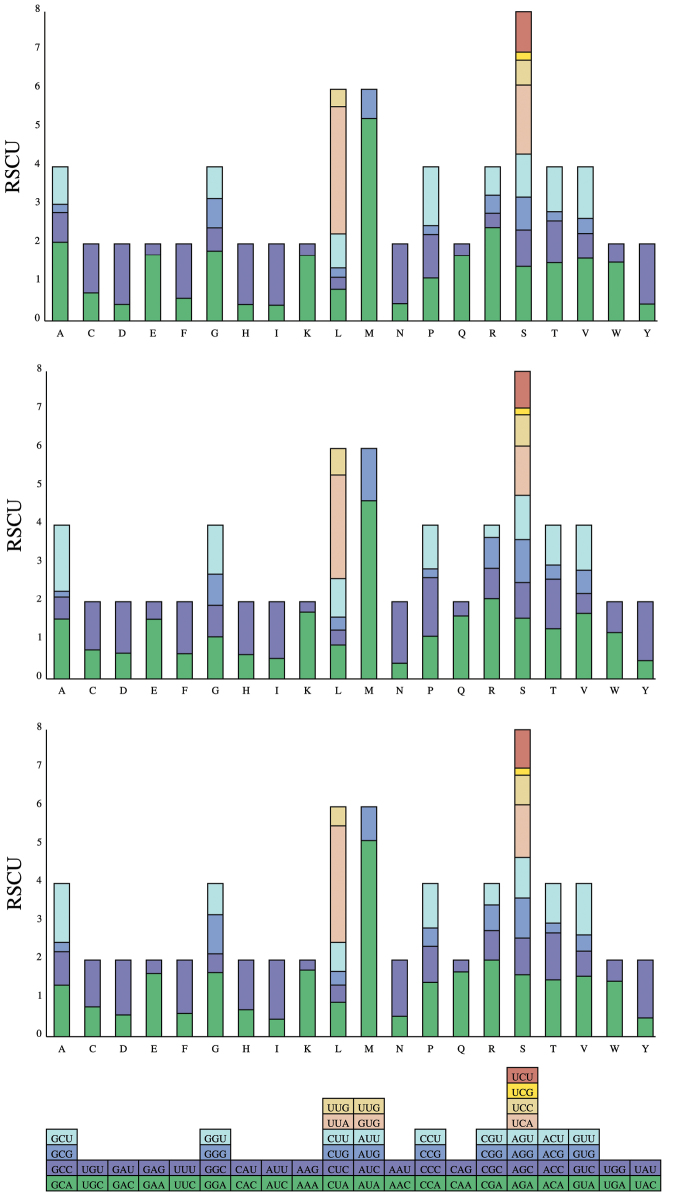
Relative synonymous codon usage (RSCU) in the mitogenomes of *Hygialativentris*, *H.bidentata*, and *H.opaca*. A: Ala; C: Cys; D: Asp; E: Glu; F: Phe; G: Gly; H: His; I: Ile; K: Lys; L: Leu; M: Met; N: Asn; P: Pro; Q: Gln; R: Arg; S: Ser; T: Thr; V: Val; W: Try; Y: Tyr.

The three mitogenomes of *Hygia* encoded 5,437, 5,674, and 5,674 amino acids, respectively (Suppl. material 1: table S1). The RSCU values were summarized. Among them, the most frequent codons were AAU (N), AAA (K), UAU (Y), AUA (M), AUU (I), UAA (L), and UUU (F) (Fig. 3), all of which were composed of only A or U, which may play an important role in the A+T bias throughout the mitogenome.

**Figure 4.**
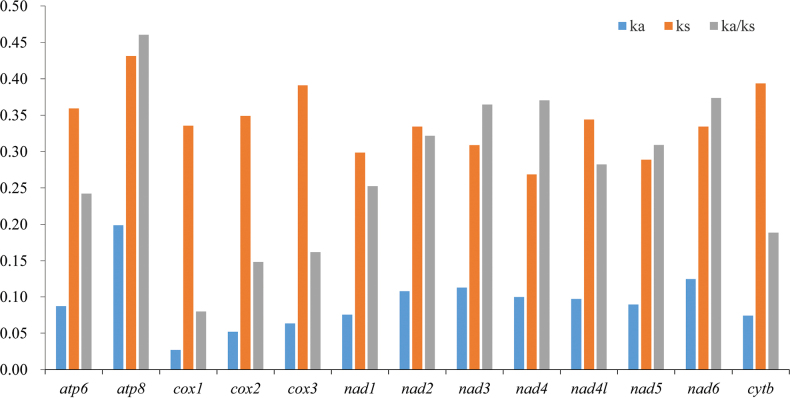
Evolution rate of each protein-coding gene (PCG) of the three *Hygia* mitogenomes.

The ratios of the nonsynonymous (Ka)/synonymous (Ks) substitution rates (Ka/Ks) for the 13 PCGs from the three *Hygia* species were calculated and used to estimate the evolutionary rate (Hurst 2002). We found that the Ka/Ks ratios of all 13 PCGs were lower than one, ranging from 0.079–0.460, which indicates that they were under purifying selection. Among them, the average Ka/Ks ratio of *cox1* was the lowest (0.079), consistent with other insect groups such as *Pyrrhocoris* (Hemiptera) (Zhang et al. 2019b) and *Eysarcoris* (Hemiptera) (Li et al. 2021). Because of this characteristic, *cox1* is often used for DNA barcoding. In contrast, *atp8* had the highest Ka/Ks ratio (0.460), which makes it useful for analyzing intra-species relationships. The Ka/Ks ratios of *cox1*, *cox2*, *cox3*, and *cytb* were all lower than 0.200, which indicated that these species bore strong purification selection and evolutionary constraints (Fig. 4).

### ﻿Transfer and ribosomal RNAs

**Figure 5.**
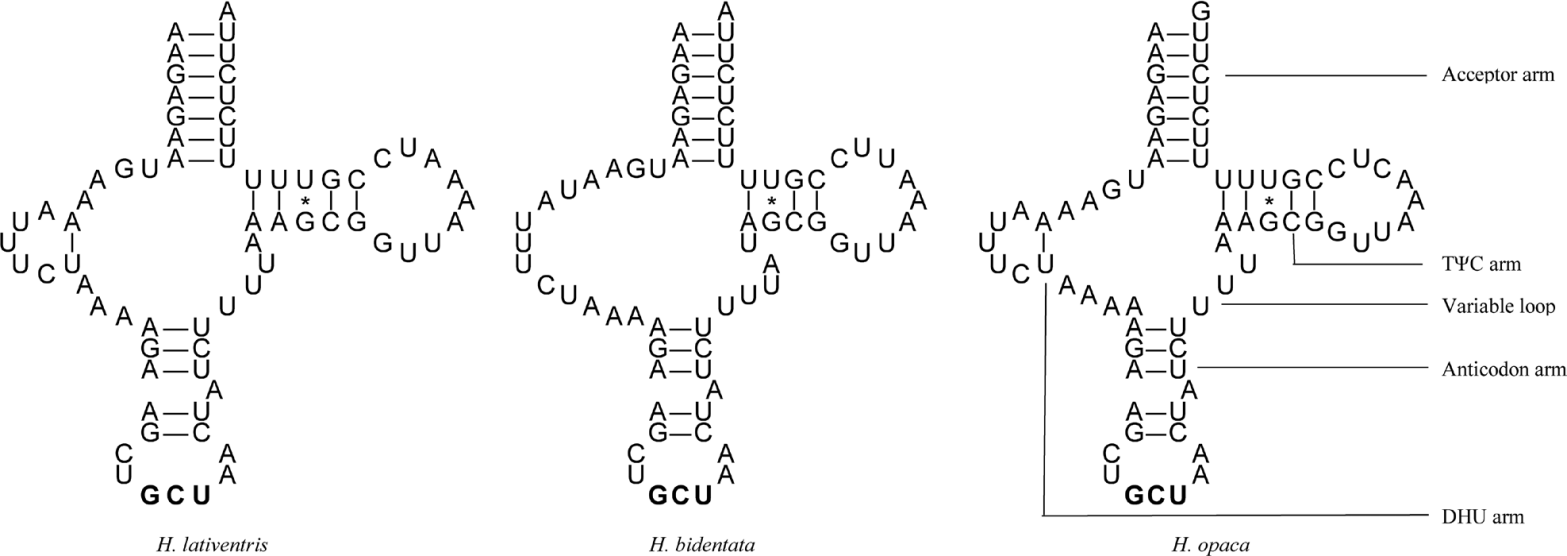
Predicted secondary structure of *trnS1* (AGC) of the three *Hygia* species.

The complete mitogenomes of the three *Hygia* species contained 22 tRNA genes. Fourteen tRNA genes (*trnI*, *trnM*, *trnW*, *trnL2*, *trnK*, *trnD*, *trnG*, *trnA*, *trnR*, *trnN*, *trnS1*, *trnE*, *trnT*, and *trnS2*) were located on the J-strand, and eight tRNA genes (*trnQ*, *trnC*, *trnY*, *trnF*, *trnH*, *trnP*, *trnL1*, and *trnV*) were located on the N-strand. The 22 tRNA gene sequences were 62–74 bp in length. The entire tRNA region was 1,453 bp, 1,459 bp, and 1,455 bp, respectively (Suppl. material 1: tables S1, S2). The tRNAs secondary structures had a typical clover structure, except for the *trnS1* (AGC) of *H.bidentata*, which had a reduced dihydrouridine (DHU) arm that formed a simple loop. Moreover, one type of mismatched base pair (U-G) was found in the tRNA secondary structure in the mitogenomes of the three species (Fig. 5, Suppl. material 1: figs S1–S3).

Similar to the published pentatomid mitogenomes, two ribosomal RNA genes (*rrnL* and *rrnS*) were encoded on the N‐strand in the mitogenomes. The lengths of *rrnL* in *H.lativentris*, *H.bidentata*, and *H.opaca* were 1,277 bp, 1,272 bp, and 1,275 bp, respectively, all located between tRNA‐Leu (TAG) and tRNA‐Val (Suppl. material 1: table S1). The lengths of the *rrnS* were 790 bp, 790 bp, and 797 bp, respectively, all located between tRNA‐Val and the control region. The A+T contents of *rrnL* were 78.9%, 79.3%, and 78.9%, respectively, whereas those of *rrnS* were 78.9%, 79.2%, and 79.8%, respectively. Both *rrnL* and *rrnS* had a positive AT skew and a negative GC skew (Suppl. material 1: table S2).

### ﻿Control region

**Figure 6.**
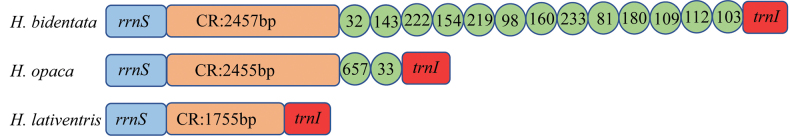
Control region in the three mitogenomes of the three *Hygia* species. The position and copy number of tandem repeats are indicated by green circles (repeat length inside). The length of the control region is indicated by the brown box (sequence size inside).

The control region is related to the origin of transcription and replication (Saito et al. 2005). The control region, or A+T-rich region, was located between *rrnS* and *trnI*. The length ranged from 1,755 to 2,457 bp. The different mitogenome lengths are mainly because of the variation in the length of the control region. The nucleotide compositions had a positive AT and a negative GC skew (Suppl. material 1: tables S1, S2). Figure 6 shows a comparison of the control regions of the three species. Two and 13 types of tandem repeat units occurred in the control region of *H.opaca* and *H.bidentata*, whereas none was detected in *H.lativentris* (Fig. 6).

### ﻿Phylogenetic analyses

**Figure 7.**
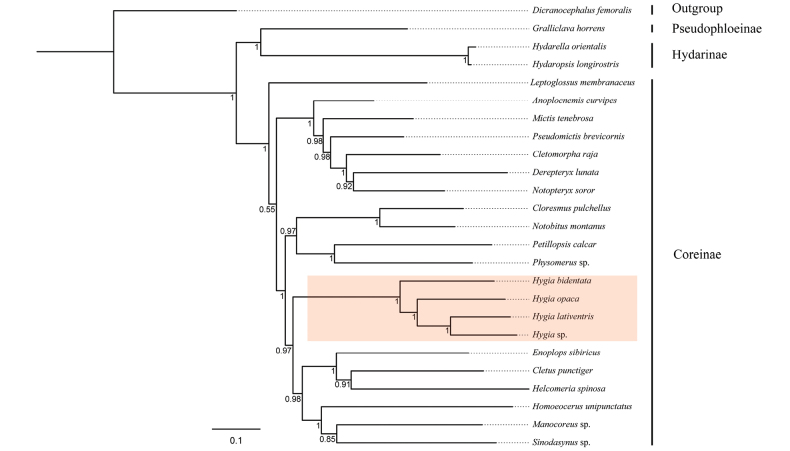
Inferred Bayesian-inference phylogenetic tree of Coreidae, based on the 13 protein-coding genes (PCGs). Numbers at the nodes indicate Bayesian posterior values.

**Figure 8.**
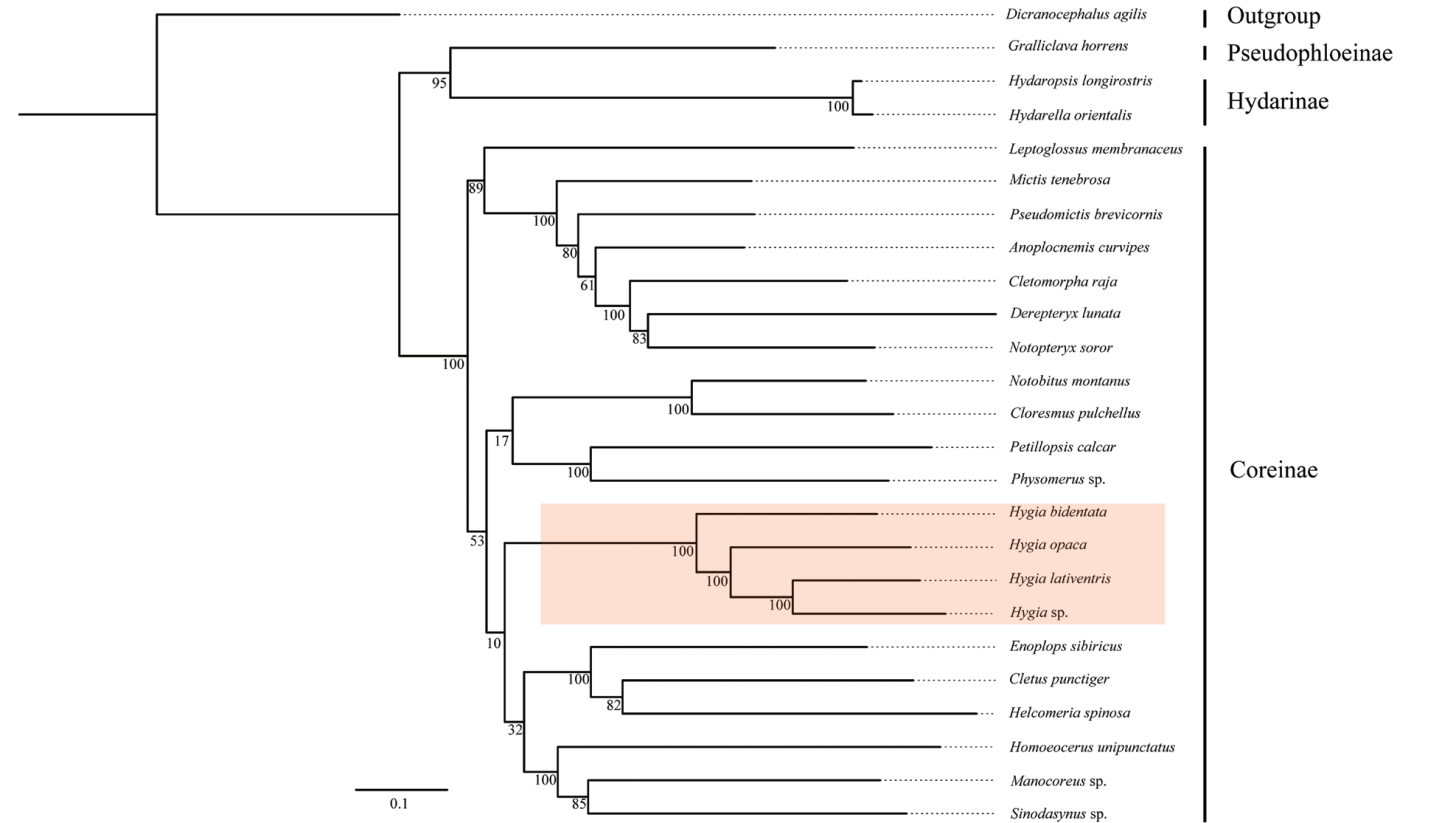
Phylogenetic tree of Coreidae inferred by IQ‐TREE based on the 13 protein-coding genes (PCGs). Numbers at the nodes indicate bootstrap values.

We performed the phylogenetic analyses using 25 mitochondrial genomes to reconstruct the relationships among the genus *Hygia* and other genera in Coreidae, which included species from three subfamilies (Figs 7, 8). Despite the high posterior probabilities but low bootstrap values, the BI and ML analyses of the dataset comprising all three codon positions and 13 PCGs all showed highly congruent tree topologies for Coreidae, except regarding the position of *A.curvipes* (Figs 7, 8). The three *Hygia* species are well grouped; *H.opaca* and *H.lativentris* are closely related. At the genus level, the analysis placed the three species of the present study in a strongly supported clade ((*H.bidentata* + (*H.opaca* + (*H.lativentris + Hygia* sp.))) (Figs 7, 8).

## ﻿Discussion and conclusions

In this study, we first described the newly sequenced mitogenomes of three *Hygia* species, *H.bidentata*, *H.lativentris*, and *H.opaca*, and found that *Hygia* mitogenome arrangements are highly conserved. The results are consistent with other published mitochondrial genomes of Hemiptera (Lee et al. 2009; Zhao et al. 2018; Zhang et al. 2019a). The mitogenome lengths range from 16,313 bp to 17,023 bp, mainly due to the different tandem repeats size of the control region. The nucleotide composition of the three species shows a strong AT preference. Moreover, the most frequent codons in the RSCU were composed of only A or U. This preference for nucleotide composition is generally thought to be caused by mutational pressures and natural selection (Hassanin et al. 2005).

The protein-coding genes started with the standard codon ATN, except for the *cox1*, which starts with a non-traditional start codon TTG. Moreover, except for *cox1*, *cox2*, and *cox3*, which used T codons—a common feature in insects—the other PCGs finish with a complete TAN codon. The stop codon TAA is more frequent than TAG in all three mitogenomes. (Zhao et al. 2018; Zhang et al. 2019b; Li et al. 2021).

The tRNAs secondary structures in *Hygia* had a typical clover structure, except for the *trnS1* (AGC) of *H.bidentata*, which had a reduced dihydrouridine (DHU) arm that formed a simple loop. This reduced DHU arm in *trnS1* is common in most metazoans (Cameron 2014). Moreover, one type of mismatched base pair (U-G) was found in the tRNA secondary structure (Pons et al. 2014).

We performed the phylogenetic analyses based on BI and ML using a dataset with the 13 PCGs, our results are generally consistent with some studies on phylogenetic analysis of the bug subfamily Coreinae (Forthman et al. 2019; Dong et al. 2022; Ye et al. 2022). Given the limited taxon sampling in our research so far, it is not possible to prove the monophyly of Coreidae. However, several previous studies using different datasets and inference methods, have confirmed that Coreidae is nonmonophyletic (Li 1997; Zhao et al. 2018; Liu et al. 2019; Forthman et al. 2019, 2020, 2022; Dong et al. 2022; Ye et al. 2022). At the same time, our study strongly supports that *Hygia* is monophyletic and formed a clade ((*H.bidentata* + (*H.opaca* + (*H.lativentris + Hygia* sp.))). However, the classification system of Coreidae is extensive and complicated. In the present study, we used too few species; therefore, the monophyly of the genus *Hygia* cannot be confirmed. Currently, the Coreidae phylogeny is based on past non-cladistic morphological studies and molecular data (UCEs and mitogenome). Although these phylogenomic analyses of Coreoidea are still non-consensual, these findings offer some potential for a few clades. Therefore, further systematic work is required to discern this relationship.

In conclusion, three new mitogenomes of the genus *Hygia* were sequenced and compared in this study. The results show the monophyly of *Hygia* and the relationships within Coreidae. The genus *Hygia* is very diverse, but only a few species of the group were used in this analysis. Therefore, we need to analyze more groups and add more mitogenome sequences to better reconstruct and evaluate the phylogeny at different levels, including family and subfamily level relationships within the superfamily Coreoidea, by combining morphological and molecular data.

## ﻿Additional information

### Author ORCIDs

Shijun Wang https://orcid.org/0000-0002-2673-9677

Xiaofei Ding https://orcid.org/0009-0000-5519-1316

Wenbo Yi https://orcid.org/0000-0002-0963-8364

Qing Zhao https://orcid.org/0000-0003-2744-1239

Hufang Zhang https://orcid.org/0000-0003-4279-9597

### Data availability

All of the data that support the findings of this study are available in the main text or Supplementary Information.

## Supplementary materials

Supplementary material 1 Supplementary information https://doi.org/10.3897/zookeys.1179.100006.suppl1 Data type docx Explanation note **table S1.** Mitogenomic organization of *H.lativentris*, *H.bidentata*, and *H.opaca*; **table S2.** Nucleotide composition and skewness of different elements of mitogenomes of *H.lativentris*, *H.bidentata*, and *H.opaca*; **figures S1–S3.** Predicted secondary cloverleaf structure of tRNA of *H.lativentris*, *H.bidentata*, and *H.opaca*, respectively. https://binary.pensoft.net/file/903317This dataset is made available under the Open Database License (http://opendatacommons.org/licenses/odbl/1.0/). The Open Database License (ODbL) is a license agreement intended to allow users to freely share, modify, and use this Dataset while maintaining this same freedom for others, provided that the original source and author(s) are credited.Shijun Wang, Xiaofei Ding, Wenbo Yi, Wanqing Zhao, Qing Zhao, Hufang Zhang
