# Supplementary material for: ﻿Comparative mitogenomic analysis of three bugs of the genus Hygia Uhler, 1861 (Hemiptera, Coreidae) and their phylogenetic position
Source: Zookeys. 2023 Sep 8;1179:123–38. doi: 10.3897/zookeys.1179.100006 (PMC10504634; doi:10.3897/zookeys.1179.100006)
Supplement: Supplementary material 1 — Supplementary information [file zookeys-1179-123_article-100006__-s001.docx]

**Supplementary material**

**Table S1.** Mitogenomic organization of *H. lativentris*, *H. bidentata*, and *H. opaca*

| **Position Intergenic Codon** | | | | | | | |
| --- | --- | --- | --- | --- | --- | --- | --- |
| **Gene** | **From** | **To** | **Size nucleotides** | | **Start** | **Stop** | **Strand** |
| *H. lativentris*/ *H. bidentata* / *H. opaca* | | | | | | |  |
| *trnI* | 1/1/1 | 64/65/63 | 64/65/63 | -3/-3/-3 |  |  | J |
| *trnQ* | 62/63/61 | 130/131/129 | 69/69/69 | -1/-1/-1 |  |  | N |
| *trnM* | 130/131/129 | 197/198/196 | 68/68/68 | 1/1/1 |  |  | J |
| *nad2* | 199/200/198 | 1200/1204/1202 | 1002/1005/1005 | -2/-2/-2 | ATG/ATG/ATG | TAA/TAA/TAA | J |
| *trnW* | 1199/1203/1201 | 1264/1268/1266 | 66/66/66 | -8/-8/-8 |  |  | J |
| *trnC* | 1257/1261/1259 | 1318/1324/1320 | 62/64/62 |  |  |  | N |
| *trnY* | 1319/1325/1321 | 1383/1386/1385 | 65/62/65 | 1/1/2 |  |  | N |
| *cox1* | 1385/1388/1388 | 2918/2921/2921 | 1534/1534/1534 |  | TTG/TTG/TTG | T/T/T | J |
| *trnL2* | 2919/2922/2922 | 2983/2987/2986 | 65/66/65 |  |  |  | J |
| *cox2* | 2984/2988/2987 | 3662/3666/3665 | 679/679/679 |  | ATA/ATA/ATA | T/T/T | J |
| *trnK* | 3663/3667/3666 | 3735/3740/3738 | 73/74/73 | -1/-1/-1 |  |  | J |
| *trnD* | 3735/3740/3738 | 3797/3802/3800 | 63/63/63 |  |  |  | J |
| *atp8* | 3798/3803/3801 | 3956/3958/3959 | 159/156/159 | -7/-7/-7 | ATC/ATT/ATT | TAA/TAA/TAA | J |
| *atp6* | 3950/3952/3953 | 4621/4623/4624 | 672/672/672 | -1/-1/-1 | ATG/ATG/ATG | TAA/TAA/TAA | J |
| *cox3* | 4621/4623/4624 | 5407/5409/5410 | 787/787/787 |  | ATG/ATG/ATG | T/T/T | J |
| *trnG* | 5408/5410/5411 | 5470/5473/5475 | 63/64/65 |  |  |  | J |
| *nad3* | 5471/5474/5476 | 5824/5827/5829 | 354/354/354 | -/-1/-1 | ATT/ATT/ATT | TAA/TAG/TAG | J |
| *trnA* | 5825/5827/5829 | 5889/5893/5897 | 65/67/69 | 3/4/3 |  |  | J |
| *trnR* | 5893/5898/5901 | 5955/5960/5963 | 63/63/63 | 1/2/1 |  |  | J |
| *trnN* | 5957/5963/5965 | 6023/6027/6031 | 67/65/67 | -1/-1/-1 |  |  | J |
| *trnS1* | 6023/6027/6031 | 6092/6096/6100 | 70/70/70 | -1/-1/-1 |  |  | J |
| *trnE* | 6092/6096/6100 | 6156/6160/6164 | 65/65/65 | -2/-2/-2 |  |  | J |

| **Position Intergenic Codon** | | | | | | | |
| --- | --- | --- | --- | --- | --- | --- | --- |
| **Gene** | **From** | **To** | **Size nucleotides** | | **Start** | **Stop** | **Strand** |
| *H. lativentris*/ *H. bidentata* / *H. opaca* | | | | | | |  |
| *trnF* | 6155/6159/6163 | 6220/6227/6227 | 66/69/65 | 1/1/1 |  |  | N |
| *nad5* | 6222/6229/6229 | 7934/7941/7941 | 1713/1713/1713 | 1/1/1 | ATG/ATG/ATG | TAG/TAA/TAG | N |
| *trnH* | 7963/7943/7943 | 7997/8005/8005 | 62/63/63 | -1/-1/3 |  |  | N |
| *nad4* | 7997/8005/8008 | 9313/9321/9319 | 1317/1317/1311 | -7/-7/-7 | ATG/ATG/ATG | TAA/TAA/TAA | N |
| *nad4l* | 9307/9315/9313 | 9591/9602/9597 | 285/288/285 | 2/2/2 | ATT/ATT/ATT | TAA/TAA/TAA | N |
| *trnT* | 9594/9605/9600 | 9657/9666/9662 | 64/62/63 |  |  |  | J |
| *trnP* | 9658/9667/9663 | 9722/9732/9728 | 65/66/66 | 2/2/2 |  |  | N |
| *nad6* | 9725/9735/9731 | 10207/10220/10213 | 483/486/483 | -1/-1/-1 | ATT/ATA/ATA | TAA/TAA/TAA | J |
| *cytb* | 10207/10220/10213 | 11343/11356/11349 | 1137/1137/1137 | -2/-2/-2 | ATG/ATG/ATG | TAG/TAG/TAG | J |
| *trnS2* | 1134211355/11348 | 11411/11424/11417 | 70/70/70 | 21/24/19 |  |  | J |
| *nad1* | 11433/11449/11437 | 12353/12366/12360 | 921/918/924 |  | ATT/ATT/ATT | TAA/TAA/TAG | N |
| *trnL1* | 12354/12367/12361 | 12420/12435/12426 | 67/69/66 |  |  |  | N |
| *rrnL* | 12421/12436/12427 | 13697/13707/13701 | 1277/12721275/ |  |  |  | N |
| *trnV* | 13698/13708/13702 | 13768/13776/13770 | 71/69/69 |  |  |  | N |
| *rrnS* | 13769/13777/13771 | 1455814566/14567 | 790/790/797 |  |  |  | N |
| *NCR* | 14559/14567/14568 | 16313/17023/17022 | 1755/2457/2455 |  |  |  | J |

**Table S2.** Nucleotide composition and skewness of different elements of mitogenomes of *H. lativentris*, *H. bidentata*, and *H. opaca*

| **Regions** | **Size (bp)** | **T(U)%** | **C%** | **A%** | **G%** | **A+T%** | **AT**-**skew** | **GC**-**skew** |
| --- | --- | --- | --- | --- | --- | --- | --- | --- |
| *H. lativentris, H. bidentata and H. opaca* | | | | | | | | |
| Whole genome | 16313/17023/17022 | 33.9/33.2/32.5 | 13.4/13.5/14.3 | 42.9/44.1/43.6 | 9.7/9.2/9.6 | 76.8/77.3/76.1 | 0.12/0.14/0.15 | -0.16/-0.19/-0.20 |
| Protein-coding genes | 11043/11046/11043 | 43.7/43.3/43.1 | 10.8/10.9/11.3 | 33.5/34.0/33.4 | 12.1/11.8/12.1 | 77.2/77.3/76.5 | -0.13/-0.12/-0.13 | 0.07/0.04/0.03 |
| Protein-coding genes-J | 6807/6810/6810 | 38.5/37.8/37.4 | 12.5/13.1/13.6 | 37.5/38.0/37.8 | 11.4/11.1/11.2 | 76.0/75.8/75.2 | -0.01/0.00/0.00 | -0.05/-0.08/-0.10 |
| Protein-coding genes-N | 4236/4236/4233 | 52.0/52.1/52.4 | 7.8/7.4/7.7 | 27.0/27.5/26.3 | 13.1/13.0/13.6 | 79.0/79.6/78.7 | -0.32/-0.31/-0.33 | 0.25/0.37/0.28 |
| tRNA genes | 1453/1459/1455 | 36.1/36.2/36.1 | 12.2/12.1/12.9 | 41.6/41.1/40.5 | 10.0/10.6/10.5 | 77.7/77.3/76.6 | 0.07/0.06/0.06 | -0.10/-0.07/-0.10 |
| tRNA genes-J | 926/928/929 | 36.2/36.2/37.2 | 10.6/10.3/10.1 | 41.7/41.5/40.9 | 11.6/12.0/11.7 | 77.9/77.7/78.1 | 0.07/0.07/0.05 | 0.05/0.08/0.07 |
| tRNA genes-N | 527/523/525 | 36.1/36.7/34.1 | 15.0/15.5/17.7 | 41.6/40.9/39.8 | 7.4/8.4/8.4 | 77.7/77.6/73.9 | 0.07/0.05/0.08 | -0.34/-0.30/-0.36 |
| rRNA genes | 2067/2062/2072 | 33.2/32.0/33.0 | 13.2/16.2/13.2 | 45.8/47.3/48.3 | 7.9/7.4/7.5 | 79.0/79.3/79.3 | 0.159/0.194/0.167 | -0.252/-0.376/-0.277 |
| Control region | 1755/2457/2455 | 31.9/30.0/27.2 | 18.9/16.2/19.3 | 39.8/46.0/43.7 | 9.4/7.8/9.8 | 71.7/76.0/70.9 | 0.11/0.21/0.23 | -0.34/-0.35/-0.33 |


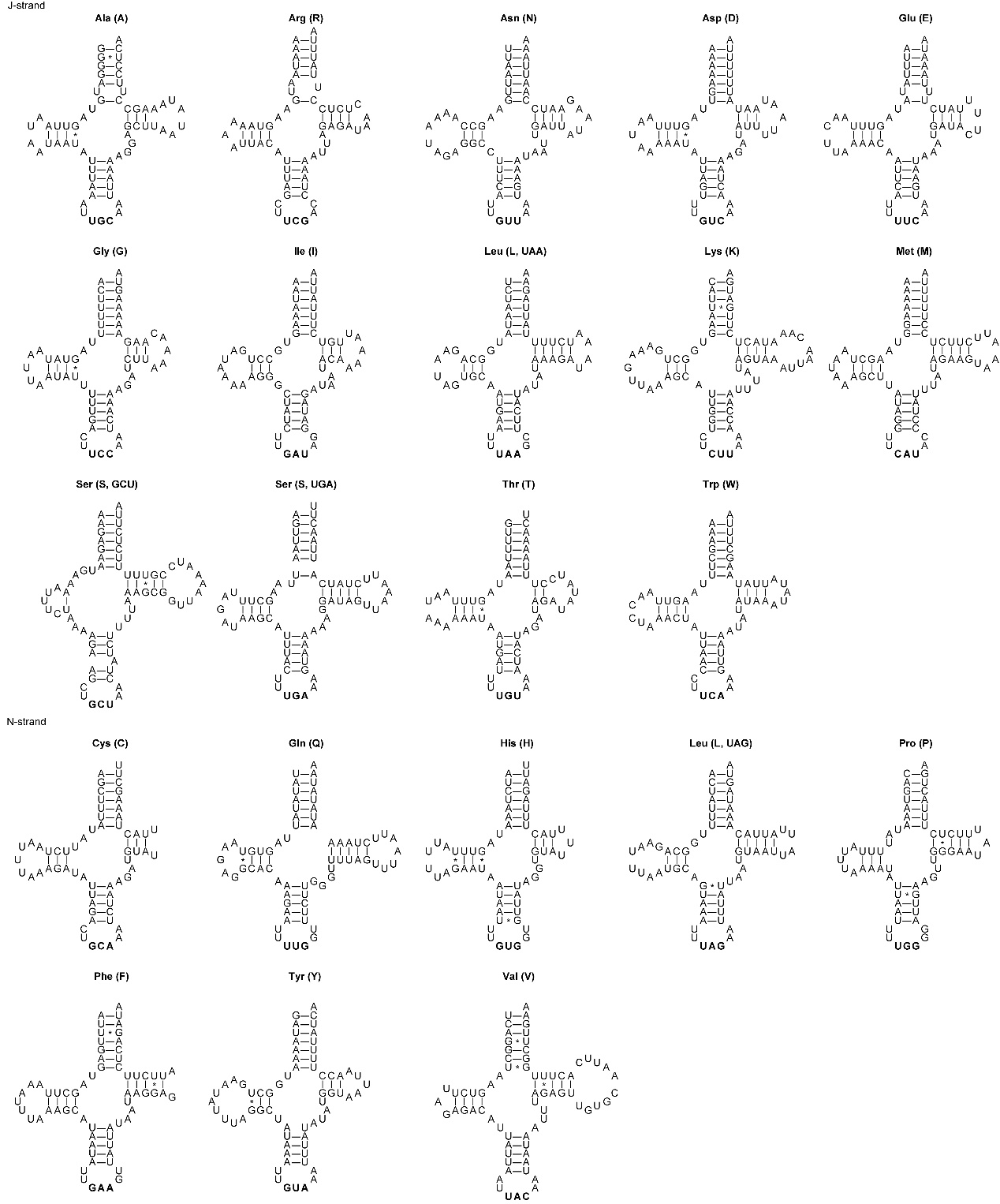


**Figure S1** Predicted secondary cloverleaf structure of tRNA of *H. lativentris*.


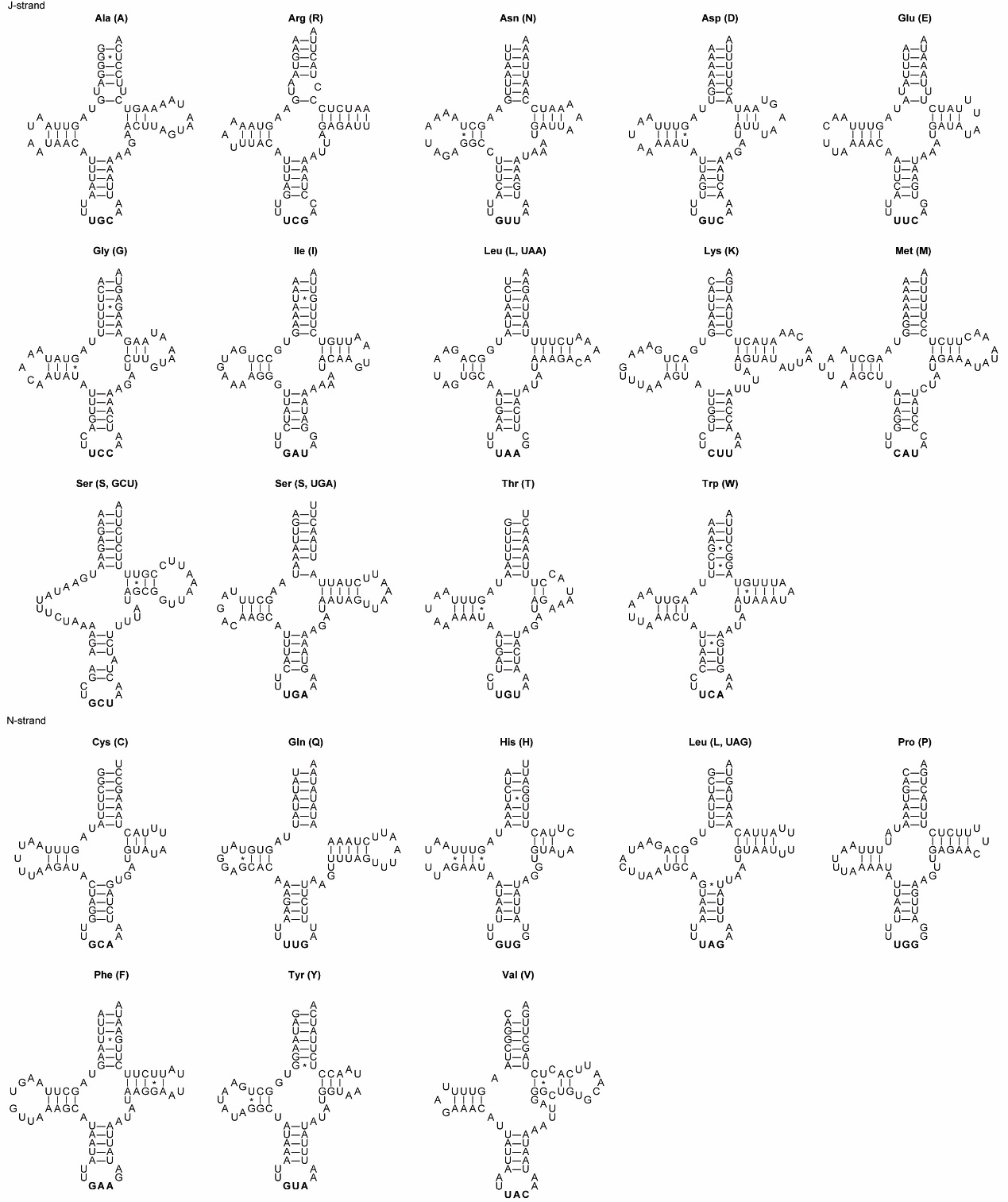


**Figure S2** Predicted secondary cloverleaf structure of tRNA of *H. bidentata*.


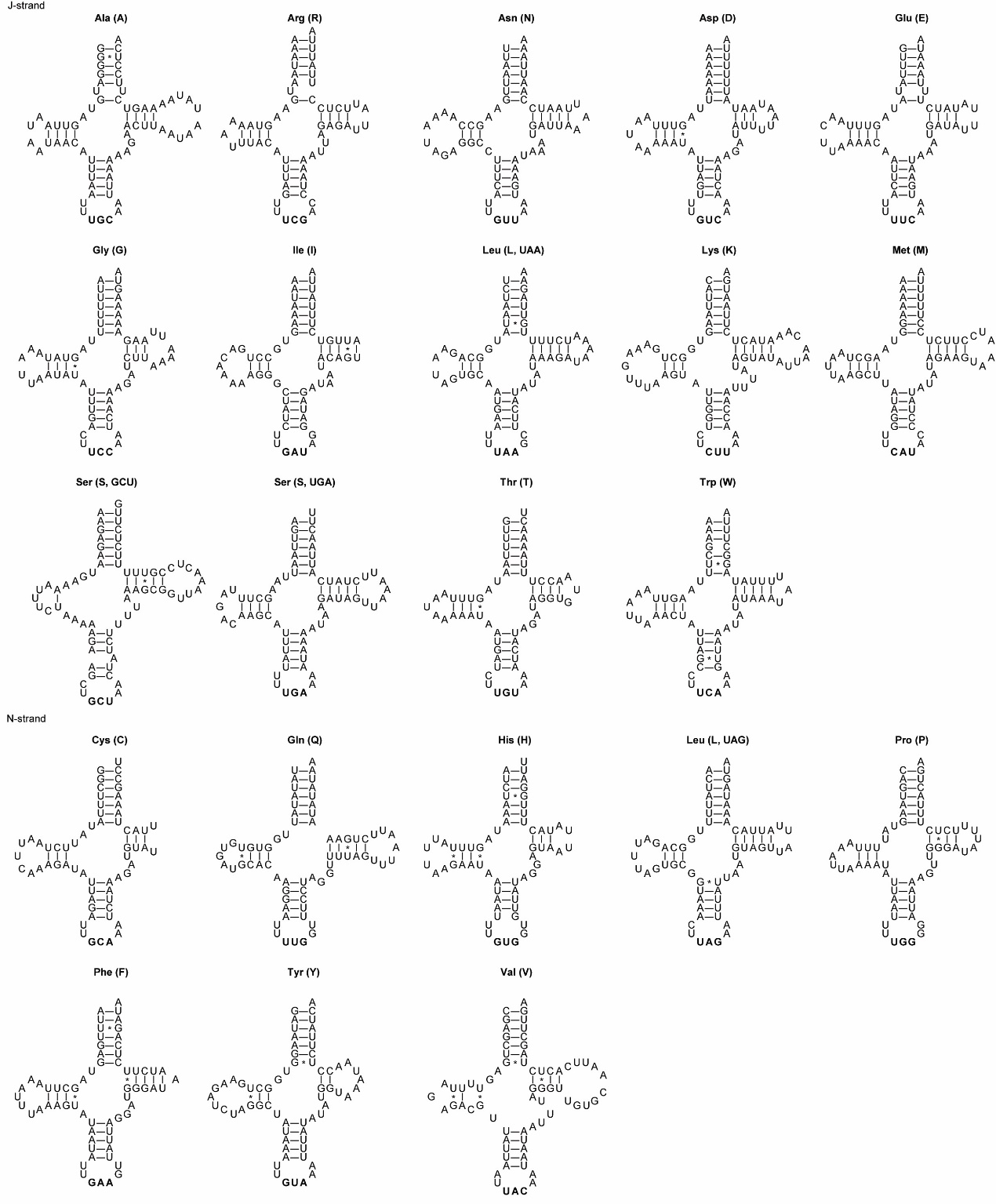


**Figure S3** Predicted secondary cloverleaf structure of tRNA of *H. opaca*.
